# Supplementary material for: WordCommentsAnalyzer: A windows software tool for qualitative research
Source: F1000Res. 2018 Sep 4;7:536. Originally published 2018 May 3. [Version 2] doi: 10.12688/f1000research.14819.2 (PMC6137411; doi:10.12688/f1000research.14819.2)
Supplement: Supplementary file 3 [file f1000research-7-17614-s0002.tgz › b2a79496-4ada-47c4-9682-a99ce867836d.zip!/Eight Iranian Health Journals/Yafteh _52.docx]

© Yafteh  . This work is licensed under CC BY 4.0 (https://creativecommons.org/licenses/by/4.0/).

Volume & Issue: دوره 8 - شماره 1

Title: بررسی اثر انالاپریل بر نوروپاتی دیابتی در موش صحرائی

http://yafte.lums.ac.ir/article-1-1046-fa.html

مقدمه: نوروپاتی یکی از عوارض دیابت است که از اهمیت خاصی برخوردار است . کاهش سرعت هدایت عصب که هم در نوروپاتی های دیابتی در انسان و هم در مدلهای حیوانی دیابت بوجود می آید به عنوان یک شاخص مهم نوروپاتی دیابتی در نظر گرفته می شود. در این تحقیق اثر داروی انالاپریل بر کاهش سرعت هدایت عصب، تعداد مویرگهای اندونوریوم و ضخامت غشای پایه مویرگها در عصب سیاتیک حرکتی در موش صحرائی دیابتیک بررسی شده است. مواد و روش ها: این تحقیق یک مطالعه تجربی است که در آن از 50 سر موش صحرائی نر (gr450-300)استفاده شد. موشها به پنج گروه 10تائی (کنترل، شم، دیابتی، پیشگیری و درمان) تقسیم شدند. القای دیابت بوسیله تزریق زیر جلدی آلوکسان (mg/kg140) انجام گرفت. وزن بدن و گلوکز خون حیوانات در روز شروع آزمایشات و در پایان هر هفته اندازه گیری شد. به گروههای شم و پیشگیری از روز شروع آزمایشات وبه گروه درمان ازپایان هفته پنجم به بعد به مدت چهار هفته‌روزانه mg/kg 5 انالاپریل به صورت خوراکی تجویز شد. سرعت هدایت عصب درعصب سیاتیک حرکتی در روزپایان آزمایشات اندازه‌گیری شد ، سپس تعداد مویرگهای داخل عصب و وضعیت ضخامت غشای ‌پایه مویرگها در گروههای مختلف بررسی گردید. نتیجه گیری: نتایج این تحقیق نشان می دهد که تجویز خوراکی داروی انالاپریل که یک مهار کننده آنزیم مبدل آنژیوتانسین می باشد ، می تواند نقش مؤثری در پیشگیری و درمان نوروپاتی دیابتی داشته باشد. یافته ها: دیابت به مدت 5 هفته باعث کاهش سرعت هدایت عصب به میزان 45% گردید‌ (05/0p<). در گروه پیشگیری تجویز خوراکی وروزانه انالاپریل به مدت 5 هفته توانست از کاهش سرعت هدایت عصب در موشهای دیابتی شده به میزان 62% جلوگیری کند (05/0 p<) . تجویز روزانه انالاپریل به موشهای گروه درمان باعث بهبود کاهش سرعت هدایت عصب به میزان 66% (05/0p<) و همچنین افزایش تعداد مویرگهای اندونوریوم به میزان 67% نسبت به گروه دیابتیک گردید(05/0p<). داروی انالاپریل بر سرعت هدایت عصب و تعداد مویرگهای اندونوریوم در موشهای غیر دیابتی اثری نداشت.

Title: یسه تأثیر دکستروز 10% با اکسی توسین40 واحد در لیتردرجمع شدن رحم پس از سزارین

http://yafte.lums.ac.ir/article-1-1047-fa.html

مقدمه: باتوجه به اهمیت عارضه خونریزی رحمی بعد از سزارین و بنا به تناقضات اثر داروهای مختلف بر روی آن ، مطالعه‌ای به منظور مقایسه اثر دکستروز 10% با اکسی توسین 40 واحد در لیتر در سفت وجمع شدن رحم در زایشگاه شهید شبیه‌خوانی شهرستان کاشان در سال 1384 انجام شد. مواد و روش ها: این مطالعه به صورت کار آزمایی بالینی دو سوکور به منظور مقایسه تأثیردو گروه دارویی دکستروز 10% با اکسی توسین 40 واحد در لیتر درجمع وسفت شدن رحم درزنان حامله‌ای که تحت سزارین الکتیو قرار می گرفتند وبصورت تخصیص تصادفی به دو گروه دریافت‌کننده انفوزیون 200 میلی لیتر دکستروز 10% (60نفر) و دریافت‌کننده انفوزیون 40 واحد در لیتر اکسی توسین (60نفر)تقسیم شده بودند، انجام شد. اطلاعات لازم با روش مشاهده جمع آوری شد و نتایج با آزمون آماری کای - اسکور مورد تجزیه و تحلیل آماری قرار گرفت. نتیجه گیری: تزریق دکستروز 10% در جمع وسفت شدن رحم بعد از سزارین موثرتر از دوز اکسی توسین 40 واحد در لیتر بود. به ویژه هرچه تعدادزایمان فرد بیشتر باشد این تأثیرواضح تراست لذا می توان جهت کنترل خونریزی وکمک به جمع وسفت شدن رحم بعد ازسزارین بدلیل عوارض دوز بالای اکسی توسین ، از دکستروز 10% به جای اکسی توسین 40 واحد در لیتر استفاده کرد. یافته ها: نتایج حاصل ازاین مطالعه نشان داد که گروههای مورد مطالعه به لحاظ عوامل مؤثر بر پاسخ‌دهی به داروها مثل سن و وزن اختلاف معنی‌داری ندارند. همچنین نتایج تحقیق نشان داد که در گروه دارویی دکستروز 10%، میزان پاسخ دهی یعنی جمع وسفت شدن رحم بطور قابل توجهی دارای تفاوت معنی دار در مقایسه با گروه دارویی اکسی توسین 40 واحد در لیتر بود (05/0 p<). انفوزیون د کستروز 10% درزنانیکه بیشتر از یک زایمان داشته اند موثرتراز زنان زایمان اول بود و روش دارویی اکسی توسین اضافی 40 واحد در لیتر در زنان زایمان اول موثرتربود (05/0 p<).

Title: شناسایی سویه‌هایO157:H7 مولد شیگا توکسین اشریشیا کلی (STEC) جدا شده از نمونه‌های مدفوع وادرار بیماران به روشMultiplex PCR

http://yafte.lums.ac.ir/article-1-1048-fa.html

مقدمه: عفونت با سویه‌های مولد شیگا توکسین اشریشیاکلی(STEC) علاوه بر ایجاد اسهال با عوارض وخیم ومرگ آوری چون سندروم اورمی‌همو لیتیک(HUS) همراه است. این در حالی است که روشهای تشخیصی این باکتریها مشکل بوده و اطلاع دقیقی از میزان بروز بیماری در ایران نیست.این مطالعه با هدف بررسی سویه‌های مولد شیگا توکسین اشریشیا کلی جدا شده از نمونه‌های ادرار و مدفوع به روش PCR در مبتلایان، انجام گرفت. مواد و روش ها: در این مطالعه که یک پژوهش بنیادی-کاربردی است، از بین 500 نمونه‌های جمع آوری شده 100 نمونه که با تستهای فیزیولوژیک بیشترین امکان وجود انواع O157 درآنها وجود داشت، انتخاب شد وپس از استخراج DNA از کلنی های E.coliحاصل از کشت،واکنش Multiplex PCR برای ژنهای stx2 وstx1 انجام گرفت و محصول آن بر روی ژل آگارز الکتروفورز گردید و پس از رنگ آمیزی با اتیدیوم بروماید مورد بررسی قرار گرفت. یافته ها: در بین 100 سویه جدا شده (مشکوک بهSTEC) تنها 3 مورد (3 درصد کل نمونه‌ها) از نظر ژنstx مثبت بودند که هر سه مورد مربوط به سویه‌های stx2 بود. دو مورد آن مربوط به نمونه مدفوع ویک مورد مربوط به نمونه ادرار بود. نتیجه گیری: اگر چه ظاهراّ شیوع عفونت با سویه‌های STEC درایران بالا نیست؛ اما به دلیل بروز عوارض وخیمی چون HUS و کولیت هموراژیک از یک سو وعدم شناسایی این سویه‌ها با روش های مرسوم در آزمایشگاه‌های کشور از سوی دیگر، بکارگیری روشهای مولکولی به منظور شناسایی موارد مشکوک،حداقل در مراکز طبی کودکان، ضروری به نظر می‌رسد.

Title: پایش شیوع گواتر و میزان ید ادرار در دانش‌آموزان 10-7 ساله استان لرستان در سال 1380

http://yafte.lums.ac.ir/article-1-1049-fa.html

مقدمه: کمبود ید و اختلال‌های ناشی از آن به عنوان یک مشکل عمده بهداشتی ـ تغذیه‌ای در ایران از سال‌ها قبل شناخته شده است. استان لرستان یکی از مناطقی است که در مطالعات قبل از یدرسانی دارای شیوع بالای گواتر آندمیک بوده است. در ایران برنامه کنترل و پیشگیری از بروز این اختلال‌ها از جمله تولید و توزیع نمک یددار از سال 1368 به مرحله اجرا درآمده است. به منظور پایش برنامه کشوری مبارزه با کمبود ید در سال 1380 این بررسی در دانش‌آموزان 7 تا 10 ساله استان لرستان انجام شد. مواد و روش ها: طی یک بررسی توصیفی ـ مقطعی از طریق نمونه‌گیری تصادفی تعداد 1200 دانش‌آموز (به تعداد مساوی دختر و پسر) انتخاب شدند. شیوع گواتر از طریق معاینه بالینی انجام و طبق تقسیم‌بندی سازمان جهانی بهداشت درجه‌بندی شد. نمونه ادرار از یک دهم نمونه‌ها اخذ گردید و اندازه‌گیری ید ادرار به روش هضم انجام شد. نتیجه گیری: یافته‌های این بررسی نشان می‌هد که در مقایسه با بررسی کشوری سال 1375 درصد گواتر در دانش‌آموزان کاهش معنی‌داری داشته و ید ادرار دانش‌آموزان استان لرستان همچنان در حد مطلوب سازمان جهانی بهداشت می‌باشد. بنابر نتایج این پژوهش استان لرستان در زمره مناطق "عاری از کمبود ید "محسوب می‌شود. یافته ها: درصد کلی گواتر در دانش‌آموزان این استان 8/7 درصد، به ترتیب در دختران و پسران 8/7 و 7/7 درصد بود (P=NS). میانه ید ادرار در جمعیت مورد مطالعه 17 میکروگرم در دسی‌لیتر بود. در 7/85 درصد موارد ید ادرار بیشتر از 10 میکروگرم در دسی‌لیتر بود و سطح ید ادرار کمتر از 5 میکروگرم در دسی‌لیتر در 7/1 درصد موارد وجود داشت. در هیچ یک از موارد ید ادرار کمتر از 2 میکروگرم در دسی‌لیتروجود نداشت. تفاوتی در میزان ید ادرار بین دو جنس و نیز بین دانش‌آموزان شهر و روستا مشاهده نشد(P=0.NS).

Title: تاثیر میدان مغناطیسی زمین بر عملکرد دستگاههای تصویر برداری پزشکی هسته ای (SPECT)

http://yafte.lums.ac.ir/article-1-1050-fa.html

مقدمه: دستگاه های تصویربرداری پزشکی هسته ای نسبت به تغییرات میدان مغناطیسی در محیط حساس هستند. فونومولتی پلایرها وظیفه تقویت سیگنالهای کم انرژی نور و تبدیل آنها به جریان های الکتریکی از طریق تکثیر الکترونی به عهده دارند. میدانهای مغناطیسی ضعیف می توانند سبب تغییراتی در یکنواختی و قدرت تفکیک سیستم تصویربرداری شوند. در این مطالعه سعی شده است تا اثر میدان مغناطیسی محیط بر عملکرد سیستم های تصویربرداری پزشکی هسته ای به هنگام چرخش دوربین در زوایای مختلف مورد بررسی قرار گیرد. مواد و روش ها: در این تحقیق سه دستگاه تصویربرداری مختلف SPECT با جهت گیری نصب متفاوت مورد بررسی قرار گرفتند. با قرار دادن چشمه نقطه ای و خطی در مقابل دوربین ، جابجایی قله طیف انرژی پرتو دارو، قدرت تفکیک و خطی بودن آن به هنگام چرخش دوربین در میدان مغناطیسی زمین در زوایای مختلف بررسی گردید. این محاسبات در دو حالت خاموش و روشن بودن دستگاه خنک کننده (کولر گازی) در فاصله 2 متری دوربین گاما، تکرار شد. با انجام آزمون t-test بین گروهها نتایج مورد بررسی قرار گرفت. نتیجه گیری: میدانهای مغناطیسی در اطراف یک سیستم تصویربرداری پزشکی هسته ای می تواند در عملکرد صحیح آن اختلال ایجاد کند. یافته ها: نتایج آزمون t-testنشان داد که در هر سه دستگاه SPECT مورد بررسی مقدار جابجایی فوتوپیک و اعوجاج در قدرت تفکیک و خطی بودن در زوایای مختلف بصورت معنی دار تغییر می کند (01/0p<).

Title: ارزیابی قدرت اثر بخشی ضد عفونی کننده ها و آنتی سپتیک ها ی مورد مصرف در بیمارستانهای آموزشی

http://yafte.lums.ac.ir/article-1-1051-fa.html

مقدمه: عوامل عمده گسترش میکروارگانیسمهای ایجاد کننده عفونتهای بیمارستان می تواند عدم استفاده صحیح از ضد عفونی کننده ها و آنتی سپتیک ها ی مورد مصرف در بیمارستانها باشد. هدف از مطالعه حاضر ارزیابی قدرت اثر بخشی ضد عفونی کننده ها و آنتی سپتیک ها ی مورد مصرف در بخش های پانسمان، تزریقات، اتاقهای عمل و آشپزخانه بیمارستانهای آموزشی همدان بود. مواد و روش ها: در این مطالعه توصیفی ـ تحلیلی ـ مقطعی، مجموعاً 742 کشت میکروبی قبل و بعد از ضدعفونی از بخشهای پانسمان – تزریقات، اتاقهای عمل و آشپزخانه دو بیمارستان آموزشی مورد بررسی قرار گرفت. مواد ضد عفونی کننده مورد استفاده عبارت بودند از : هالامید، به آسا، کرئولین، فرمالدئید، گلوتارآلدئید، کلرهکزیدین، هایژین، ساولن، الکل 70 درجه و اشعه UV. اثر بخشی مواد ضد عفونی کننده تهیه شده در بیمارستان ها با مواد ضد عفونی کننده تهیه شده در آزمایشگاه میکروب شناسی دانشکده پزشکی بر روی استافیلوکوک اورئوس (ATCC 25923) و E.coli (ATCC 25922) به روش انتشار در آگار مورد ارزیابی و مقایسه قرار گرفت. اطلاعات در پرسشنامه وارد شده و با استفاده از آزمون آماری کای دو و مجموع رتبه ای ویلکاکسون (من – ویتنی) و نرم افزار spss مورد آنالیز قرار گرفت. نتیجه گیری: میانگین میزان آلودگی قبل از ضد عفونی و بعد از ضد عفونی در دو بیمارستان اختلاف معنی داری داشت (001/0p=) که نشاندهنده کاهش نسبی میکروارگانیسمهای بیمارستانی بعد از ضد عفونی کردن است. اما با توجه به اختلاف معنی دار نتایج اثر بخشی ضد عفونی کننده ها تهیه شده در بیمارستان ها و دانشکده پزشکی، پیشنهاد می گردد، تهیه ضد عفونی کننده ها مورد مصرف در بیمارستانها و نحوه استفاده آنها در بخشهای مختلف با نظارت کارشناسان بهداشت محیط بیمارستان صورت گیرد. یافته ها: از مجموع 742 نمونه کشت داده شده در دو بیمارستان، 308 مورد (6/41%) کشت مثبت بدست آمد. میانگین میزان آلودگی قبل از ضد عفونی 1/50% و بعد از ضد عفونی 8/33% تعیین شد. از مجموع 308 مورد کشت مثبت در دو بیمارستان، 2/56% باکتری گرم منفی و 8/43% باکتری گرم مثبت بدست آمد. بیشترین میزان آلودگی (2/58%) مربوط به آشپزخانه و کمترین آن (6/18%) مربوط به اتاق های عمل بود مؤثرترین ماده ضد عفونی تست شده در دو بیمارستان عبارت بودند از فرمالدئید 8%، گلوتارآلدئید 2%، کلرهکزیدین 1%، ساولن 2/3% و کم اثرترین آنها عبارت بود از: محلول به آسا 1%، الکل70% و کرئولین 5/2%. در مورد اثر بخشی اشعه UV برای اتاق پانسمان و تزریقات، میزان درصد آلودگی هوای اتاق قبل از ضد عفونی 4/51% و بعد از ضد عفونی 6/48% بود که اختلاف معنی داری بدست نیآمد. اثر بخشی ضد عفونی کننده ها تهیه شده در بیمارستانهای مورد پژوهش با نتایج بدست آمده در آزمایشگاه میکروب شناسی دانشکده پزشکی اختلاف معنی دار نشان داد.

Title: بررسی حساسیت و ویژگی دیلاتاسیون و کورتاژ در تشخیص خونریزی غیر طبیعی رحمی

http://yafte.lums.ac.ir/article-1-1052-fa.html

مقدمه: خونریزی رحمی غیرطبیعی (AUB) یکی از شایعترین مشکلات پزشکی در قلمرو بیماریهای زنان محسوب می‌شود. یکی از روشهای تشخیص انجام دیلاتاسیون و کورتاژ (D&C) می‌باشد که براساس نتایج حاصل از آن ممکن است اقدامات درمانی قطعی‌تر همچون هیسترکتومی صورت گیرد. هدف از این مطالعه، تعیین حساسیت و ویژگی D&C در AUB بود. نتیجه گیری: براساس نتایج این بررسی D&C روشی نسبتاً بی‌خطر و ارزان و در دسترس برای بررسی اغلب موارد AUB می‌باشد. توصیه می‌شود در مواردی که علیرغم وجود تخمک‌گذاری، خونریزی غیرطبیعی ادامه دارد و یا در موارد شک بالینی به ضایعات آندومتریال، هیستروسکپی مدنظر باشد. یافته ها: نتایج مطالعه نشان داد 3/75% بیماران ساکن روستا و 6/70% آنها بی‌سواد بودند. اغلب بیماران، سه حاملگی یا بیشتر را تجربه کرده بودند و فقط 4/2% در گروهی قرار داشتند که کمتر از 3 حاملگی داشتند. میانگین سنی بیماران 25/47 سال بود، حداقل سن 26 و حداکثر 78 سال بود. روش پیشگیری از حاملگی در اکثریت بیماران مورد مطالعه در زمان هیسترکتومی بستن لوله‌های رحمی بود (2/41%). علت هیسترکتومی در حدود 1/94% بیماران ادامه خونریزی غیرطبیعی علیرغم درمان طبیعی بود. حساسیت روش D&C در تشخیص خونریزی غیر طبیعی رحمی 26/78 درصد و ویژگی آن 16/79 درصد بود. مواد و روش ها: در این مطالعه توصیفی نتایج هیستوپاتولوژیک حاصل از D&C و هیسترکتومی با هم مقایسه شدند. تعداد 85 بیمار مبتلا به AUB که تحت D&C و سپس هیسترکتومی (بدلیل عدم پاسخ به درمان طبی یا گزارش پاتولوژیست دال بر پاتولوژی آندومتر غیرقابل درمان طبی) قرار گرفته بودند، وارد مطالعه شدند. مطالعه در بیماران مبتلا به AUB مراجعه کننده به بیمارستان بعثت در طـی سالهای 1381 لغایت 1383 (سه سال) صـورت گرفت. داده های لازم با استفاده از مشاهدات پاتولوژیست نتایج مربوط D&C و هیسترکتومی به دست آمده و در چک‌لیست ثبت می شد. این داده ها با استفاده از آمار توصیفی، تست x2 و نرم افزار SPSS تجزیه و تحلیل شد.

Title: تاثیر احتمالی سمیت سلولی عنصرروی بر سلولهای رده لنفوئیدی راجی (Raji) باتکنیک فلورسانس

http://yafte.lums.ac.ir/article-1-1053-fa.html

مقدمه: عنصر روی( Zn ) درحفظ سلامتی انسان ودر ساختار و عملکرد سیستم ایمنی نقش بارزی دارد. این مطالعه به منظور بررسی تاثیر احتمالی سمیت سلولی عنصر روی بر سلولهای- B (راجی) در شرایط آزمایشگاهی انجام شد. نتیجه گیری: ترکیبات روی بررده سلولی راجی دارای اثر سمیت سلولی وابسته به دوز بوده واحتمالا بتوان از آن در آینده در تنظیم سیستم ایمنی استفاده نمود. یافته ها: آنالیز داده ها نشان داد که بین میزان زنده ماندن و رشد سلول ها در گروههای آزمون و شاهد تا غلظت‌ 100میکرومولار در ساعات 12 تا 72 اختلاف معنی داری وجود ندارد؛ اما در 200میکرومولار تا 500 میکرومولار بعد از 12و24و... ساعت انکوباسیون ,این پارامترها درگروه آزمون نسبت به گروه شاهدکاهش معنی دار یافت ( 05/0 < p ). مواد و روش ها: این مطالعه تحلیلی-توصیفی وبنیادی با استفاده از تکنیک کشت سلولی رده لنفوئیدی راجی در شرایط آزمایشگاهی در مجاورت غلظت های مختلف روی در زمانهای متفاوت(12تا72 ساعت) در دمای 37درجه سانتیگراد و5 درصد Co2 انکوبه گردید . آنگاه میزان زنده ماندن ورشد سلول ها با رنگ آمیزی فلورسانس ) 10 میکرو لیتر از مخلوط اکریدین اورنج و اتید یوم برو ماید) بررسی شد. نتایج بدست آمده با برنامه نرم افزار Spss (آزمون آنالیز واریانس و دانت) مورد تجزیه وتحلیل قرارگرفت.

Title: رابطه بین الکل، عوامل غذایی و سرطان در زیر قسمتهای آناتومیک روده بزرگ؛ یک مطالعه اکولوژیک

http://yafte.lums.ac.ir/article-1-1054-fa.html

مقدمه: سرطان روده بزرگ چهارمین سرطان شایع از لحاظ میزان وقوع در جهان است. بعضی تفاوت ها بین وقوع سرطان در سه زیر قسمت آناتومیک روده بزرگ از نظر ارتباط با عوامل خطرزا برای سرطان وجود دارد؛ اما اکثر تحقیقات رابطه بین عوامل غذایی به عنوان عوامل خطر و سرطان در زیر قسمتهای آناتومیک روده بزرگ را (روده راست، روده چپ و رکتال) بررسی نکرده اند. بنابراین مطالعه اکولوژیک حاضر رابطه بالقوه بین بعضی عوامل غذایی و وقوع سرطان در این زیر قسمتهای اناتومیک را بررسی می کند. مواد و روش ها: اطلاعات موجود درباره میزان وقوع سرطان روده بزرگ به تفکیک زیر قسمتهای آناتومیک و همچنین سرانه مصرف سالانه بعضی عوامل غذایی مشکوک و الکل در ایجاد این نوع سرطان برای 32 کشور مختلف دنیا طی سالهای 1988 تا 1992 میلادی به دست آمد. رابطه بین الکل و عوامل غذایی و سرطان در زیر قسمتهای آناتومیک روده بزرگ توسط روش آماری رگرسیون خطی مورد آنالیز قرار گرفت. نتیجه گیری: تحقیق و بررسی بروز سرطان در زیر قسمتهای آناتومیک روده بزرگ به طور جداگانه می تواند ما را در شناخت علل این سرطان و در نتیجه راههای پیشگیری از آن کمک نماید. یافته ها: بعد از انجام مدل رگرسیون چند متغیره مشخص گردید که مصرف گوشت رابطه معنی داری با بروز سرطان روده راست در مردان دارد) 02/0 = p). بر عکس مصرف الکل یک عامل مهم و معنی دار برای بروز سرطان روده چپ در مردان (03/0= p ) و برای سرطان رکتال در زنان (04/0 = p) و مردان (01/0 = p) است.

Title: میزان شیوع و گستردگی ناامنی غذایی از دو بعد گرسنگی عیان و گرسنگی پنهان در منطقه اسد آبادی تبریز

http://yafte.lums.ac.ir/article-1-1056-fa.html

مقدمه: ناامنی غذایی و گرسنگی می تواند علاوه بر تاثیر بر سلامت جسمی، تبعات اجتماعی و روانی نیز به همراه داشته باشد. از اینرو تامین امنیت غذایی برای جامعه به عنوان یکی از اهداف کلان برنامه ریزیهای توسعه اقتصادی و اجتماعی است. مطالعه حاضر با هدف تعیین میزان شیوع و گستردگی ناامنی غذایی از دو بعد گرسنگی عیان و پنهان در شمال غرب تبریز به عنوان نمونه ای از وضعیت ناامنی غذایی در استان آذربایجان شرقی به اجرا درآمد. مواد و روش ها: این مطالعه مقطعی- توصیفی بر روی 300 نفر( دامنه سنی 78-18 سال) که از این تعداد 168 نفر زن و 132 نفر مرد بودند انجام گرفت. برای انتخاب افراد مورد مطالعه نمونه گیری به روش نمونه گیری آسان انجام شد. ابتدا پرسشنامه ای که شامل مشخصات فردی( سن، جنس، وزن و قد) افراد مورد مطالعه بود، کامل شد و سپس اطلاعات مربوط به مواد غذایی توسط پرسشنامه یادآمد خوراک 24 ساعت گذشته که شامل ثبت غذای سه روز هفته( یک روز تعطیل و دو روز وسط هفته) بود تهیه گردید. شاخص گرسنگی عیان مصرف ناکافی انرژی و شاخص گرسنگی پنهان مصرف ناکافی مواد مغذی کلیدی تعیین گردید و سپس با برنامه کامپیوتری Nutrition III آنالیز داده ها انجام گرفت. نتیجه گیری: بر اساس نتایج حاصل، ناامنی غذایی در جامعه شیوع بالایی دارد که با توجه به تاثیر آن در سلامت افراد، برنامه ریزی برای حل مشکل و همچنین دادن اطلاعات صحیح در مورد دریافت مواد غذایی متعادل، متنوع و کافی لازم و ضروری به نظر می رسد. یافته ها: میزان شیوع ناامنی غذایی از بعد گرسنگی عیان 26 درصد و از بعد گرسنگی پنهان 6/41 درصد بود. در افراد با گرسنگی عیان، 100 درصد افراد کمبود کلسیم، 8/94 درصد کمبود ریبوفلاوین، 3/74 درصد کمبود پروتئین و 4/56 درصد کمبود ویتامینA داشتند. در افراد با گرسنگی پنهان، 6/89 درصد کمبود کلسیم، 6/77 درصد کمبود ریبوفلاوین، 2/19 درصد کمبود پروتئین و 6/9 درصد کمبود ویتامین A داشتند. به طور کلی فقط 3/32 درصد افراد مورد مطالعه از لحاظ تامین هر پنج ماده مغذی کلیدی یعنی انرژی، پروتئین، ویتامینA، ریبوفلاوین و کلسیم امن بودند.

Title: مقایسه نمره ”آپگار دقیقه“ 5 و 10 نوزادان متولد شده به روش زایمان بی درد به روش تسکین وریدی و سزارین

http://yafte.lums.ac.ir/article-1-1057-fa.html

مقدمه: زایمان بی درد به روش تسکین وریدی، روشی است که در آن با استفاده از تزریق وریدی داروهای ضد درد و آرامبخش به مادر، درد و اضطراب فرآیند زایمان را کاهش می یابد. در این روش سلامت نوزاد مورد توجه است. با استفاده از سیستم آپگار می توان سلامت نوزاد متولد شده را بررسی کرد. این مطالعه با هدف بررسی آپگار دقایق 5 و 10 نوزادان متولد شده به روش زایمان بی درد با تسکین داخل وریدی و سزارین انجام شد. مواد و روش ها: در این مطالعه مداخله ای تعداد 60 بیمار در دو گروه 30 نفری که به صورت سرشماری از بین خانم های بارداری که جهت زایمان طبیعی و سزارین انتخابی، انتخاب شده بودند، مورد بررسی قرار گرفتند. زایمان بی درد به روش تزریق وریدی کتامین و میدازولام و زایمان سزارین به روش بیهوشی عمومی انجام شد و نمره آپگار دقایق 5 و 10 در هر دو گروه در پرسشنامه ای که به همین منظور آماده شده بود، ثبت گردید. سپس با استفاده از آزمون آماری U من ویتنی اختلاف آماری بین دو گروه را بررسی شد. نتیجه گیری: مطالعه نشان داد با وجود مصرف داروهای ضد درد و ضد اضطراب و همچنین با وجود عبور جنین از کانال زایمانی، نوزادان گروه زایمان بی درد نسبت به نوزادان گروه سزارینی دچار کاهش نمره آپگار نشدند که این مسئله نشان می دهد که زایمان بی درد به روش تسکین داخل وریدی، یک زایمان ایمن می باشد. یافته ها: در این مطالعه از لحاظ سن و پاریته اختلاف معنی داری بین دو گروه وجود نداشت. 90% نوزادان گروه زایمان بی درد در دقیقه 5 و تمامی نوزادان در دقیقه 10 نمره آپگار 9 و 10 داشتند. در گروه سزارین در دقیقه پنجم، 20% نوزادان و در دقیقه دهم، 10% نوزادان نمره آپگار 7 و 8 داشتند. آزمون من ویتنی اختلاف آماری معنی داری در نمره آپگار 5 و 10 دو گروه نشان نداد. در این مطالعه تمامی نوزادان نمره آپگار 7 تا 10 داشتند. میانگین فاصله زمانی آخرین تزریق دارو تا زایمان بین دو گروه اختلاف معنی داری داشت که در زایمان بی درد فاصله به مراتب بیشتر بود (83/11 دقیقه در مقابل 5/5 دقیقه).

Title: میزان آگاهی، نگرش و عملکرد دختران دانش آموز دبیرستانی در مورد بهداشت دوران قاعدگی

http://yafte.lums.ac.ir/article-1-1058-fa.html

مقدمه: یکی از وقایع مهم زندگی افراد دوران نوجوانی و بلوغ است. عامل شناسایی بلوغ واقعی در دختران شروع اولین قاعدگی است. علیرغم اینکه قاعدگی در زندگی دختران یک واقعه غیر قابل انکار است کمتر در مورد آن و عواملی که بر آن تاثیر می گذارند مطلب نوشته می شود. در این راستا پژوهش فوق با هدف تعیین نیازهای آموزشی (آگاهی ـ نگرش ـ عملکرد ) دختران دبیرستانی در موررد بهداشت دوران بلوغ ( قاعدگی ) در مدارس خرم آباد انجام گرفت . مواد و روش ها: در این مطالعه توصیفی ـ تحلیلی تعداد 700 دانش آموز دختر سال اول دبیرستان شهر خرم آباد بصورت تصادفی سیستماتیک انتخاب شدند. ابزار گرد آوری داده ها پرسشنامه شامل مشخصه های فردی ، اطلاعاتی پیرامون قاعدگی و سوالات آگاهی ، نگرش و عملکرد بود که توسط پژوهشگر و پرسشگر آموزش دیده تکمیل شد . روایی و پایایی پرسشنامه با استفاده از آزمون مجدد و اعتبار محتوا تعیین شد . اطلاعات جمع آوری شده با استفاده از نرم افزار SPSS تجزیه و تحلیل شدند. یافته ها: یافته ها نشان داد میانگین سنی نمونه ها 8/14 سال بود. 52% دارای قاعدگی نامنظم که ( 7/40 % ) دارای طول مدت دوره قاعدگی 7-5 روز بودند . 1/94% دارای اطلاعات قبلی بودند مهمترین و اولین منبع اطلاعات (1/71% ) از طریق مادر بوده است. 50% نمونه ها دارای آگاهی ضعیف بودند . نگرش 1/30% افراد مورد مطالعه نسبت به قاعدگی و علایم آن منفی بود که عملکرد آنها در این مورد با نگرش آنها توافق داشت . 9/75% دارای دیسمنوره بودند . اکثریت افراد مورد مطالعه در دوران قاعدگی دچار اختلالات خلقی ، رفتاری ( اضطراب ، ترس ) شده بودند . تجزیه و تحلیل آماری ارتباط معنا داری بین آگاهی ،نگرش ، عملکرد ومنابع کسب اطلاعات، تحصیلات والدین و شغل مادر ( 001/0 = P ) نشان داد . نتیجه گیری: بر اساس نتایج حاصله از مطالعه حاضر لزوم ارائه آموزش بهداشت دوران قاعدگی ( بلوغ ) به دختران، معلمین و مادران خصوصاً در مورد نقش تغذیه ، ورزش ، کاهش عوامل استرس زا ، رعایت نکات بهداشتی ، همچنین برنامه ریزی جهت انجام ورزشهای منظم در مدارس، بازنگری در کتب دبیرستانی و گنجاندن موضوعات بهداشت دوران بلوغ در کتاب های درسی ضروری به نظر می رسد.

Title: گزارش دو مورد هتروزیگوسیتی دو گانه تالاسمی بتا و هموگلوبین S در بیماران تالاسمی ماژور استان لرستان

http://yafte.lums.ac.ir/article-1-1061-fa.html

مقدمه: وجود همزمان دو ناهنجاری مختلف در یک ژن، هتروزیگوسیتی دو گانه نام دارد. یکی از معمول ترین انواع این هتروزیگوسیتی ،همراهی تالاسمی و بیماری سلول داسی شکل است که تالاسمی سلول داسی شکل نامیده می شود. هدف از انجام این طرح جستجوی انواع هموگلوبینوپاتیها در بیماران تالاسمی ماژور استان لرستان بوده است. نتیجه گیری: از مجموع 65 بیمار تالاسمی ماژور استان لرستان، دو بیمار مبتلا به هتروزیگوسیتی دو گانه تالاسمی بتا و هموگلوبین S بودند. با توجه به این موضوع انجام مطالعات بیشتر به خصوص در زمینه های ژنتیک برای بیماران تالاسمی و والدین آنان ضروری است. یافته ها: از میان 65 بیمار تالاسمی ماژور، والدین دو تن از آنان دارای هموگلوبین S بودند. بنابراین احتمال ابتلای فرزندان آنان به هتروزیگوسیتی دو گانه تالاسمی بتا و هموگلوبین S وجود داشت. در بررسیهای ژنتیک، جهش های شناخته شده این ژن در هر دو کروموزوم شماره 11 حامل ژن بتا گلوبین بیماران وجود نداشت. مواد و روش ها: در این مطالعه سرشماری 65 بیمار تالاسمی ماژور استان لرستان دارای پرونده و مراجعه کننده به بیمارستان شهید مدنی خرم آباد به همراه والدینشان تحت آزمایشات CBC و الکتروفورز هموگلوبین قرار گرفتند. نمونه DNA بیمارانی که والدینشان دارای هموگلوبینوپاتی بودند، جهت پی گیری امکان ابتلا به هتروزیگوسیتی دو گانه با استفاده از پرایمرهای مخصوص جهش های ژنی ، توسط روش PCR مورد آزمایش قرار گرفت.

Title: مطالعه آثار فوتوالکترون تراپی برروی سلول های سرطانی انسانی

http://yafte.lums.ac.ir/article-1-1059-fa.html

مقدمه: سرطان یکی از بیماریهای پیچیده ای است که با وجود تلاش بسیار برای درمان قطعی آن هنوز در اغلب کشورها یکی از مهمترین عوامل مرگ و میر محسوب می شود. رادیوتراپی یکی از روشهای اصلی درمان این بیماری است. سیستم فوتوالکترون تراپی یک روش جدید رادیوتراپی است که دستگاه مخصوص آن توسط مجریان این طرح جهت درمان طیف وسیعی از سرطانها طراحی و ساخته شده است. هدف از اجرای این طرح ، رسم منحنی های بقاء سلول های سرطانی انسانی در محیط های In vitro با روش فوتوالکترون تراپی و روش رادیوتراپی معمولی و اندازه گیری بازدهی درمان و بهره فوتوالکترون تراپی آنها بوده است.مواد و رش ها: روش مطالعه از نوع توصیفی و اندازه گیری اثر سیستم درمانی جدید( فوتوالکترون تراپی) بر روی سلولهای سرطانی بوده است. این اندازه گیری با استفاده از رسم منحنی های بقاء در دو شرایط فوتوالکترون تراپی و رادیوتراپی معمول صورت پذیرفت. منحنی های بقاء تعداد سلولهای کشته شده به ازای مقادیر مختلف پرتو را نشان می دهد. برای رسم این منحنی ها شش نوع از سلول های سرطانی انسانی در دسته های ده تایی (ده بار تکرار) در چهار گروه با دسته بندی زیر مورد آزمایش قرار گرفتند و میانگین نتایج شمارش ها( ده بار) برای رسم منحنی های بقاء مورد استفاده قرار گرفتند. گروه (1): تابش با روش فوتوالکترون تراپی با دز 400 سانتی گری پرتو ایکس. گروه (2): تابش با روش رادیوتراپی معمولی با همان دز 400 سانتی گری پرتو ایکس. گروه (3): ماده مؤثر به تنهایی. گروه (4): گروه شاهد( بدون اعمال هیچ تغییری). توضیح: ماده مؤثر, ماده دارویی مخصوص فوتوالکترون تراپی است.نتیجه گیری: یافته های این تحقیق یعنی منحنی های بقای سلولهای سرطانی, در شرایط In vitro نشان می دهند که روش فوتوالکترون تراپی نسبت به تابش رادیوتراپی معمولی در شرایط کاملاً یکسان 150 برابر موثرتر یا بهره ای بیش از پانزده هزار درصد را فراهم می نماید، بنابراین فوتوالکترون تراپی یک روش بسیار با ارزش برای درمان تومور های سرطانی می باشد. یافته ها: از گروه 1 ( روش فوتوالکترون تراپی) کمتر از 1% سلولها زنده ماندند. از گروه 2 (رادیوتراپی معمولی) در حدود 78% از سلولها زنده ماندند, یعنی تقریباٌ معادل گروه شاهد. از گروه 3 (تزریق ماده مو‌‌ُثر بتنهایی) در حدود 78% از سلولها زنده ماندند (تقریباٌ معادل گروه شاهد) و از گروه 4 که گروه شاهد بود نیز در حدود 78% از سلولها زنده ماندند.

Title: بررسی ارزش سطح سرمی AFP (آلفا فیتوپروتئین) در بیماران ناقل HBS Ag برای تشخیص هپاتوسلولار کارسینوما

http://yafte.lums.ac.ir/article-1-1060-fa.html

مقدمه: عفونت HBV با (یا بدون) سیروز کبدی در اکثر مناطق جهان ا جمله ایران مهمترین ریسک فاکتورهای HCC به شمار می روند. HCC در صورتی که زودتر تشخیص داده شود پیش آگهی بسیار کمتری دارد. از مهمترین روشهای غربالگری این تومور در جامعه استفاده از سونوگرافی و اندازه گیری سطح سرمی (AFP) به صورت دوره ای است. در این مطالعه ارزش و دقت تشخیصی Alfa fetorprotein (AFP) در تشخیص هپاتوسلولار کارسینوم در افراد ناقل سالم مورد بررسی قرار گرفت.مواد و روش ها: در این مطالعه 2452 فرد ناقل HBSAg بدون شواهد سیروز کبدی یا سرطان کبدی بمدت 3 سال (1380-1378) توسط سونوگرافی و اندازه گیری AFP هر 6ماه یکبار مورد بررسی قرار گرفته اند . مردان و زنان غیر حامله ای که سطح سرمی افزایش یافته داشتند با روشهای تصویر نگاری مورد بررسی زیادتر قرار گرفته اند . نتیجه گیری: AFP به تنهایی یک روش تشخیص مفید و کارآمد جهت تشخیص سرطان کبد در بیماران ناقل HBSAg نمی باشد و دارای ارزش پیشگوئی کننده و حساسیت و اختصاصیت قابل قبولی در این بیماران نمی باشد . یافته ها: AFP بالاتر از ng/ml 20 در 32 بیمار دیده شد که در بررسیهای نهایی 13 مورد ( 40% ) سرطان کبدی HCC به روش بیوپسی و تصویرنگاری ثابت شد. افزایش تولید AFP تنها 35% بود که میزان قدرت پیشگویی یک تست جهت تشخیص هپاتوم ناکافی است.
